# Evaluation of several routine methods for fosfomycin and mecillinam susceptibility testing of Enterobacterales urine isolates

**DOI:** 10.1093/jac/dkae271

**Published:** 2024-08-19

**Authors:** C Massip, L Feletti, C V Chagneau, Y Dumont, E Maurin, A Muggeo, M Pichon, M Pompilio, F Buchler, D Halimi, D Dubois

**Affiliations:** Laboratoire de Bactériologie-Hygiène, CHU de Toulouse, Université de Toulouse Paul Sabatier, Toulouse, France; Laboratoire de Bactériologie-Hygiène, CHU de Toulouse, Université de Toulouse Paul Sabatier, Toulouse, France; Laboratoire de Bactériologie-Hygiène, CHU de Toulouse, Université de Toulouse Paul Sabatier, Toulouse, France; Digestive Health Research Institute (IRSD), National Institute of Health and Medical Research (INSERM), Université de Toulouse Paul Sabatier, National Research Institute for Agriculture, Food and the Environment (INRAE), National Veterinary School of Toulouse (ENVT), Toulouse, France; Laboratoire de Bactériologie, CHU de Montpellier, Montpellier, France; Laboratoire de Bactériologie Clinique, CHU de Clermont-Ferrand, Clermont-Ferrand, France; Université de Reims Champagne-Ardenne, INSERM, CHU de Reims, Laboratoire de Bactériologie-Virologie-Hygiène Hospitalière-Parasitologie-Mycologie, P3Cell, U 1250, Reims, France; Département des Agents Infectieux, CHU de Poitiers, Laboratoire de Bactériologie, Poitiers, France; Université de Poitiers, INSERM U1070 Pharmacology of Antimicrobial Agents and Antibiotic Resistance, Poitiers, France; Microbiology R&D, bioMérieux SA, La Balme les Grottes, France; Microbiology R&D, bioMérieux SA, La Balme les Grottes, France; Microbiology R&D, bioMérieux SA, La Balme les Grottes, France; Laboratoire de Bactériologie-Hygiène, CHU de Toulouse, Université de Toulouse Paul Sabatier, Toulouse, France

## Abstract

**Objectives:**

Performance evaluation of routine laboratory methods to determine the susceptibility of Enterobacterales urinary isolates to fosfomycin (oral administration) and mecillinam.

**Methods:**

We collected 347 Enterobacterales isolates from monomicrobial midstream urine samples from women with significant bacteriuria and leukocyturia. Mostly non-*Escherichia coli* isolates (i.e. *Klebsiella* spp., *Citrobacter koseri*, *Enterobacter cloacae* complex and *Proteus mirabilis*) were included (*n* = 298). Performance of VITEK^®^2, ETEST^®^, and disc diffusion to determine fosfomycin and mecillinam susceptibility was evaluated following International Organization for Standardization (ISO) 20776-2:2021 (or 20776-2:2007 for disc diffusion) in comparison with the agar dilution reference method.

**Results:**

For fosfomycin testing, VITEK^®^2 and ETEST^®^ were close to reaching ISO requirements (essential agreement  ≥ 90%; bias  ±30%) for *C. koseri*, *E. coli* and *P. mirabilis.* Categorical agreement (CA) and major error rates were acceptable for disc diffusion. Fosfomycin displayed lower activity against *E. cloacae* complex and *Klebsiella* spp., with MIC50 (minimum inhibitory concentration required to inhibit the growth of 50% of tested isolates) equal to the *E. coli* EUCAST breakpoint (8 mg/L). For these species, the three alternative techniques overestimated MICs and resistance, and did not meet performance criteria. For mecillinam testing of Enterobacterales isolates, apart from *P. mirabilis*, ETEST^®^ nearly fulfilled ISO requirements, and CA rates were acceptable for disc diffusion. ISO criteria were reached for *C. koseri* and *E. coli* testing with VITEK^®^2, apart from too high rates of very major errors. For *P. mirabilis,* performances were unacceptable, whatever the routine method used.

**Conclusions:**

Commercially available tests may serve as alternatives to agar dilution to assess fosfomycin (oral) and mecillinam susceptibility of Enterobacterales urinary isolates, with important interspecies variabilities. Additional studies comprising more fosfomycin- and mecillinam-resistant isolates are needed to strengthen our conclusions.

## Introduction

Urinary tract infections (UTIs) are the most common infections caused by Enterobacterales.^1^ UTIs occur most frequently in women, and more than half of adult women will be diagnosed with a UTI during their lifetime.^2^ UTIs have a substantial impact on patients’ quality of life. This impact increases after antibiotic failure.^3^

Antibiotic resistance is increasing worldwide, especially in Enterobacterales. The spread of cephalosporin- and fluoroquinolone-resistant strains is of particular concern.^1,4,5^ In addition, few antibiotics are available for oral treatment of lower UTIs.^1^ Given their limited ecological impact on the gut microbiota, fosfomycin and pivmecillinam are agents of choice in the treatment of lower UTIs.^1,6^ Moreover, large European epidemiological surveys comprising community and nosocomial isolates established that resistance rates are still low for fosfomycin and pivmecillinam (around 5% and 10%, respectively), although these antibiotics are extensively used as empirical and targeted therapy.^4,5,7,8^ Interestingly, fosfomycin and pivmecillinam remain active against most MDR pathogens, especially ESBL or carbapenem-resistant Enterobacterales.^9–11^

Treatment of lower UTIs is often empirical. However, urine culture and antimicrobial susceptibility testing are recommended for isolates from patients with recurrent UTIs or presenting with at least one risk factor, which may lead to a more severe or a more difficult-to-treat infection (e.g. abnormalities of the urinary tract, pregnancy, elderly patients with frailty criteria).^6,12^ Unfortunately, fosfomycin and mecillinam (active compound released from the prodrug pivmecillinam) susceptibility testing is complicated. According to the EUCAST, the reference standard for fosfomycin and mecillinam susceptibility testing is agar dilution (AD).^13^ Because AD is labour-intensive, it is not compatible with routine laboratory diagnostics, and fosfomycin and mecillinam susceptibility testing by disc diffusion and MIC gradient test strips are often challenging for clinical microbiologists. The presence of colonies within the inhibition zone can lead to misinterpretation.^14,15^

The VITEK^®^2 automated system performance has been evaluated for fosfomycin and mecillinam susceptibility testing, but only for *Escherichia coli.* Therefore, fosfomycin and mecillinam susceptibility testing by VITEK^®^2 is not possible for non-*E. coli* Enterobacterales isolates. Even if *E. coli* is the most prevalent member of the Enterobacterales in UTIs (prevalence rate around 70%), other species are also frequent, such as *Klebsiella pneumoniae* (10%), *Proteus mirabilis* (6%), and to a lesser extent *Enterobacter cloacae* complex*, Citrobacter koseri*, *Klebsiella oxytoca* (2%–3% for each of these three species) and *Klebsiella aerogenes* (1%).^4,5^ This lack of susceptibility testing for two key oral treatments of UTIs may result in an inappropriate or non-optimal empirical treatment.

In this study, we determined fosfomycin and mecillinam susceptibility on a large panel of Enterobacterales isolates from urines with commonly used methods including VITEK^®^2, ETEST^®^ and disc diffusion. AD was used as the reference method. However, the widely used disc diffusion method cannot be evaluated according to the latest International Organization for Standardization (ISO) criteria (ISO 20776-2:2021), because it only provides requirements for ‘MIC methods’.^16^ As a result, we chose firstly to test the performance of VITEK^®^2 and ETEST^®^ with 2021 ISO criteria *sensu stricto.*^16^ Given the strong clinical need to include disc diffusion in such performance studies, we also compared the three routine methods with AD according to former ISO criteria (e.g. categorial agreement).^17^ Finally, this study gives an overview of fosfomycin and mecillinam resistance in Enterobacterales isolates from urines in France with the AD reference method, whereas most epidemiological reports use routine susceptibility methods.^4,5,7,8^

## Material and methods

### Bacterial strains

A total of 347 non-repetitive isolates from midstream urine samples were collected retrospectively and randomly from five French University Hospitals (Clermont-Ferrand, Montpellier, Poitiers, Reims and Toulouse) from 1 April 2021 to 29 February 2022. All strains were from unique female patients with monomicrobial cultures, and significant leukocyturia and bacteriuria.^6^ Patients with urinary catheter were excluded. Fifty *P. mirabilis*, 49 *C. koseri*, 49 *K. pneumoniae*, 51 *E. cloacae* complex, 50 *K. oxytoca*, 49 *K. aerogenes* and 49 *E. coli* isolates were included. Bacterial identification was checked by MALDI-TOF MS (MALDI Biotyper, Bruker).

### Susceptibility testing

For each isolate, a 0.5 McFarland (or 1 McFarland for mucoid strain) suspension was prepared in saline from a 24 h subculture on blood agar. The same bacterial suspension was made to test fosfomycin and mecillinam susceptibility by VITEK^®^2 (N372 card, bioMérieux), disc diffusion (Mast Diagnostic) and ETEST^®^ (bioMérieux) methods. Of note, the new version of ETEST^®^ developed to optimize performance was used in this study.^15^ For disc diffusion and ETEST^®^, Mueller–Hinton (MH) agar plates (bioMérieux) were used and incubated at 35 ± 2°C for 16 to 20 h. Results were read by two independent observers. Haze-like growth and isolated colonies within the inhibition zone were ignored.^13^ Susceptibility to various antibiotics was tested with VITEK^®^2 (N372 card, bioMérieux).

The CLSI AD reference method was applied to determine fosfomycin and mecillinam MICs for each strain. Briefly, MH plates with doubling AD of fosfomycin or mecillinam concentrations were prepared and a 0.5 McFarland suspension in saline and diluted to the tenth in MH broth from each isolate was inoculated. For organizational reasons, two different bacterial suspensions from the same frozen aliquot for each strain were used for AD and commercial methods. MIC was recorded as the lowest antimicrobial concentration that completely inhibited bacterial growth after 16 to 20 h incubation at 35 ± 2°C.

For fosfomycin susceptibility testing, discs, ETESTs^®^ or MH agar (for AD) were adequately supplemented with glucose-6-phosphate as recommended.^13^

*E. coli* ATCC 25922 was used as a control strain.

### Data analysis

First, we analysed the fosfomycin and mecillinam distributions with the reference method and we evaluated the performance of VITEK^®^2 and ETEST^®^ fosfomycin and mecillinam susceptibility testing according to ISO 20776-2:2021 criteria.^16^ Essential agreement (EA) was defined as an MIC within a single 2-fold dilution of the AD MIC. ISO 20776-2:2021 requires EA to be equal to or above 90% and a difference for bias ±30%.

For ETEST^®^, fosfomycin and mecillinam results were rounded up to the next agar method dilution when MIC was between two doubling dilutions.

In a second phase, we compared the three routine methods, including disc diffusion, with ISO 20776-2:2007 criteria.^17^ Categorical agreement (CA) was defined as the percentage of interpretative results (susceptible or resistant) in agreement between the routine methods (VITEK^®^2, ETEST^®^ and disc diffusion) and AD. Discrepancies in interpretation between AD and routine methods are defined as major and very major errors. Very major errors (VMEs) occurred when a routine method gave a susceptible result, whereas the AD result was resistant. Major errors (MEs) were defined as a resistant result with a routine method when the AD result was susceptible. ISO 20776-2:2007 approves ≤3% ME,  ≤ 3% VME and ≥90% CA.

When categorization was needed, mecillinam diameters and MICs were interpreted with EUCAST breakpoints: MIC  ≤ 8 mg/L or diameter  ≤15 mm, susceptible; and MIC >8 mg/L or diameter >15 mm, resistant. In the absence of interpretative criteria for non-*E. coli* Enterobacterales, EUCAST breakpoints for oral fosfomycin were used to interpret fosfomycin susceptibility for all species: MIC ≤8 mg/L or diameter ≤24 mm, susceptible; and MIC >8 mg/L or diameter >24 mm, resistant.^13^ As the concentration range tested for fosfomycin with N372 cards is 16-128 mg/L, the 8 mg/L breakpoint for oral fosfomycin cannot be reached. Therefore, isolates with a fosfomycin MIC ≤16 mg/L with VITEK^®^2 were considered susceptible.

## Results

### Fosfomycin and mecillinam MIC distributions with the reference method

Table 1 shows fosfomycin and mecillinam MIC distributions of 347 Enterobacterales urine isolates with the AD reference method. Graphical representations for each species are presented in Figure S1 (available as Supplementary data at *JAC* Online). Rates of acquired non-susceptibility to other antibiotics determined with VITEK^®^2 (e.g. amoxicillin, fluoroquinolones, cotrimoxazole) are presented in Table S1.

**Table 1.** Fosfomycin (A) and mecillinam (B) MIC distributions of tested Enterobacterales with the agar dilution reference method^a^

A.													
Fosfomycin MIC (mg/L)	0.25	0.5	1	2	4	8		16	32	64	128	256	>512
*Citrobacter koseri*	6	27	10	3			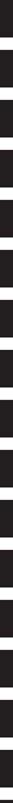	3					
*Enterobacter cloacae* complex		2	4	1	9	14		9	7	2	1		2
*Escherichia coli*	5	27	10	4	1			2					
*Klebsiella aerogenes*				6	15	14		8	5			1	
*Klebsiella oxytoca*			2	4	12	14		10	7				1
*Klebsiella pneumoniae*			1	3	12	20		7	3			2	1
*Proteus mirabilis*	4	12	15	5	2	1		1		3	2	1	4

B.															
Mecillinam MIC (mg/L)	0.06	0.12	0.25	0.5	1	2	4	8		16	32	64	128	256	>256
*Citrobacter koseri*	7	25	11	3					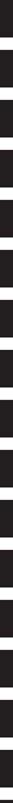					1	2
*Enterobacter cloacae* complex		4	14	13	11	2	1	3		1		1			1
*Escherichia coli*		11	18	8	7	3	2								
*Klebsiella aerogenes*			1	27	13	2		1		1	1	2			1
*Klebsiella oxytoca*			18	19	2	4	4			2			1		
*Klebsiella pneumoniae*		3	24	9	5	1	2	2		2					1
*Proteus mirabilis*		2	6	9	5	5	6	3		1	3	1	2	4	3

^a^The vertical dashed lines designate the breakpoints used (EUCAST fosfomycin breakpoint for *Escherichia coli* of 8 mg/L; EUCAST mecillinam breakpoint of 8 mg/L).

Fosfomycin was highly active against *E. coli* and *C. koseri* isolates, with only two and three resistant strains, respectively (Table 2). The MIC value for these five resistant strains was just above the cut-off concentration separating the susceptible/resistant categories. Fosfomycin displayed lower activity against *E. cloacae* complex and *Klebsiella* spp., with MIC50 (minimum inhibitory concentration required to inhibit the growth of 50% of tested isolates) equal to the EUCAST clinical breakpoint of 8 mg/L. Moreover, around half of these species isolates had MICs close to the EUCAST clinical breakpoint (i.e. with a MIC value equal to or just above the cut-off concentration between susceptible/resistant categories): 45% for *E. cloacae* complex and *K. aerogenes*, 48% for *K. oxytoca* and 55% for *K. pneumoniae.* Most *P. mirabilis* isolates (78%) were susceptible to fosfomycin, but 10 strains were highly resistant with MICs ≥64 mg/L.

**Table 2.** *In vitro* activity of fosfomycin (A) and mecillinam (B) against 347 Enterobacterales urine isolates with agar dilution reference method^a^

A. Fosfomycin				
Enterobacterales	MIC50 (mg/L)	MIC90 (mg/L)	Number of resistant strains (%)	Number of strains with MICs close to breakpoint (%)
*Citrobacter koseri*	0.5	2	3 (6)	3 (6)
*Enterobacter cloacae* complex	8	32	21 (41)	23 (45)
*Escherichia coli*	0.5	2	2 (4)	2 (4)
*Klebsiella aerogenes*	8	32	14 (29)	22 (45)
*Klebsiella oxytoca*	8	32	18 (36)	24 (48)
*Klebsiella pneumoniae*	8	32	13 (27)	27 (55)
*Proteus mirabilis*	1	128	11 (22)	2 (4)

B. Mecillinam				
Enterobacterales	MIC50 (mg/L)	MIC90 (mg/L)	Number of resistant strains (%)	Number of strains with MICs close to breakpoint (%)
*Citrobacter koseri*	0.12	0.5	3 (6)	0
*Enterobacter cloacae* complex	0.5	8	3 (6)	4 (8)
*Escherichia coli*	0.25	1	0	0
*Klebsiella aerogenes*	0.5	8	5 (10)	2 (4)
*Klebsiella oxytoca*	0.5	4	3 (6)	2 (4)
*Klebsiella pneumoniae*	0.25	4	3 (6)	4 (8)
*Proteus mirabilis*	2	256	14 (28)	4 (8)

^a^Breakpoints used: EUCAST fosfomycin breakpoint for *Escherichia coli* of 8 mg/L; EUCAST mecillinam breakpoint of 8 mg/L.

More than 90% of the strains from our collection were susceptible to mecillinam, apart from *P. mirabilis* isolates (Table 2). Of note, all *E. coli* strains were susceptible to mecillinam. MIC distribution for *P. mirabilis* was significantly wider than the other tested Enterobacterales (Table 1). Fourteen strains (28%) were resistant to mecillinam, and the MIC90 was far above the EUCAST breakpoint at 256 mg/L.

### Performance of ‘MIC methods’ with ISO 20776-2:2021 standards

#### Fosfomycin testing

The detailed results of the performance study for fosfomycin susceptibility testing with ETEST^®^ and VITEK^®^2 are presented in Tables S2 and S3, respectively.

For all the tested species, the EA rate was above the 90% threshold, except for *P. mirabilis* (88%) (Table 3). Compared with the reference method, ETEST^®^ tended to provide high MICs, as demonstrated by the too high global bias of 31.9%. Still, acceptable bias was reached for *E. cloacae* complex, *E. coli* and *P. mirabilis.* Fosfomycin MICs were largely overestimated with the ETEST^®^ method for *Klebsiella* spp. isolates, as illustrated by biases superior to 48%.

**Table 3.** Clinical performance of ETEST^®^ and VITEK ^®^ 2 for the determination of susceptibility to fosfomycin and mecillinam of 347 Enterobacterales, according to ISO 20776-2:2021 criteria^a^

		Fosfomycin		Mecillinam	
Enterobacterales	Method	EA (%)	Bias	EA (%)	Bias
*Citrobacter koseri*	ETEST^®^	47 (**96**)	34.7%	48 (**98**)	**−5.5%**
	VITEK^®^2	49 (**100**)	NC	46 (**94**)	NC
*Enterobacter cloacae* complex	ETEST^®^	47 (**92**)	**29%**	50 (**98**)	**−11.5%**
	VITEK^®^2	21 (41)	NC	36 (71)	NC
*Escherichia coli*	ETEST^®^	49 (**100**)	**−22.4%**	49 (**100**)	**18.4%**
	VITEK^®^2	49 (**100**)	NC	46 (**94**)	NC
*Klebsiella aerogenes*	ETEST^®^	46 (**94**)	61,2%	40 (82)	−34,2%
	VITEK^®^2	38 (78)	NC	12 (25)	NC
*Klebsiella oxytoca*	ETEST^®^	50 (**100**)	53,2%	44 (88)	**−8%**
	VITEK^®^2	25 (50)	NC	21 (42)	NC
*Klebsiella pneumoniae*	ETEST^®^	49 (**100**)	48,2%	48 (**98**)	**−1.5%**
	VITEK^®^2	32 (65)	NC	25 (51)	NC
*Proteus mirabilis*	ETEST^®^	44 (88)	**19.3%**	33 (69)	**−2.6%**
	VITEK^®^2	47 (**94**)	NC	19 (38)	NC

NC, not calculable.

^a^Acceptable values are in bold: essential agreement (EA) ≥90% and a difference for bias ±30%.

VITEK^®^2 met ISO requirements for fosfomycin testing of *C. koseri*, *E. coli* and *P. mirabilis* isolates, whereas EA rates were far below the 90% threshold for *E. cloacae* complex and *Klebsiella* spp. isolates (from 48.2% to 61.2%). Bias could not be calculated for VITEK^®^2 whatever the species, because the number of on-scale isolates was below 25.

#### Mecillinam testing

For two *P. mirabilis* isolates, mecillinam MIC could not be determined with ETEST^®^ because of the swarming effect. The synthesized results of the performance study for mecillinam susceptibility testing with ETEST^®^ and VITEK^®^2 are presented in Table 3. Detailed data can be found in Tables S4 and S5.

ETEST^®^ performance fulfilled the ISO criteria for *C. koseri*, *E. cloacae* complex, *E. coli* and *K. pneumoniae*. Still, EA rates were below the 90% threshold for *K. oxytoca* (88%), *K. aerogenes* (82%), and with a greater magnitude for *P. mirabilis* (69%). With the exception of *K. aerogenes*, bias was within the interval ±30% for all species.

For VITEK^®^2, ISO criteria were only reached for *E. coli* and *C. koseri* with EA rates of 94%. EA rates were particularly low for *P. mirabilis* (38%) and *K. aerogenes* (25%). Bias could not be calculated for VITEK^®^2 whatever the species, because the number of on-scale isolates was below 25.

### Comparison of the three routine laboratory methods with AD for susceptible/resistant categorization

We assessed ETEST^®^ and VITEK^®^2 performance for fosfomycin and mecillinam testing according to the latest ISO criteria (ISO 20776-2:2021).^16^ However, the disc diffusion method, which is widely used in clinical microbiology laboratories, cannot be evaluated with these criteria focusing on ‘MIC methods’. Consequently, we also compared the three commercially available tests, including disc diffusion, with AD regarding their capacity to adequately categorize fosfomycin- and mecillinam-susceptible and -resistant isolates, i.e. we evaluated the CA, VME and ME rates.

#### Fosfomycin testing

For five isolates (four *P. mirabilis* and one *K aerogenes*), the disc inhibition diameter could not be determined because the strain was too mucoid or produced too much swarming effect. The detailed results of the performance study for fosfomycin susceptibility testing with disc diffusion are presented in Table S6.

There were great performance disparities between Enterobacterales species. Two separate groups could be made, with *C. koseri*, *E. coli* and *P. mirabilis* isolates on the one hand, and *E. cloacae* complex and *Klebsiella* spp. isolates on the other hand. The three routine methods adequately categorized as susceptible or resistant to fosfomycin *C. koseri*, *E. coli* and *P. mirabilis* isolates, as demonstrated by acceptable CA or ME rates (Table 4A). However, VME rates were far above the 3% threshold. For *C. koseri*, it reached 67% (2/3 resistant strains erroneously categorized) for ETEST^®^ and disc diffusion, and 100% for VITEK^®^2 (3/3 resistant strains erroneously categorized). As regards *E. cloacae* complex and *Klebsiella* spp. isolates, none of the methods tested fulfilled acceptable criteria. The three techniques overestimated fosfomycin resistance, as demonstrated by the high ME rates.

**Table 4.** Clinical performance of the three routine methods for the determination of susceptibility to fosfomycin (A) and mecillinam (B) of 347 Enterobacterales according to ISO 20776-2:2007 criteria^a^

A. Fosfomycin				
Enterobacterales	Method	CA (%)	VME (%)	ME (%)
*Citrobacter koseri*	ETEST**^®^**	47 (**96**)	2/3 (67)	0/46 (**0**)
	VITEK**^®^**2	46 (**94**)	3/3 (100)	0/46 (**0**)
	Disc	47 (**96**)	2/3 (67)	0/46 (**0**)
*Enterobacter cloacae* complex	ETEST**^®^**	43 (84)	2/21 (10)	6/30 (20)
	VITEK^®^2	36 (71)	0/21 (**0**)	15/30 (50)
	Disc	30 (59)	0/21 (**0**)	21/30 (70)
*Escherichia coli*	ETEST^®^	48 (**98**)	1/2 (50)	0/47 (**0**)
	VITEK^®^2	47 (**96**)	2/2 (100)	0/47 (**0**)
	Disc	47 (**96**)	2/2 (100)	0/47 (**0**)
*Klebsiella aerogenes*	ETEST^®^	40 (82)	1/14 (7)	8/35 (23)
	VITEK^®^2	44 (**90**)	2/14 (14)	3/35 (9)
	Disc	13 (27)	0/13 (**0**)	35/35 (100)
*Klebsiella oxytoca*	ETEST^®^	43 (86)	1/18 (6)	6/32 (19)
	VITEK^®^2	35 (70)	2/18 (11)	13/32 (41)
	Disc	20 (40)	0/18 (**0**)	30/32 (94)
*Klebsiella pneumoniae*	ETEST^®^	35 (71)	1/13 (8)	13/36 (36)
	VITEK^®^2	38 (78)	1/13 (8)	10/36 (28)
	Disc	15 (31)	1/13 (8)	33/36 (92)
*Proteus mirabilis*	ETEST^®^	50 (**100**)	0/11 (**0**)	0/39 (**0**)
	VITEK^®^2	48 (**96**)	2/11 (18)	0/39 (**0**)
	Disc	42 (**91**)	1/10 (10)	3/36 (8)

B. Mecillinam				
Enterobacterales	Method	CA (%)	VME (%)	ME (%)
*Citrobacter koseri*	ETEST^®^	49 (**100**)	0/3 (**0**)	0/46 (**0**)
	VITEK^®^2	46 (**94**)	3/3 (100)	0/46 (**0**)
	Disc	47 (**96**)	2/3 (67)	0/46 (**0**)
*Enterobacter cloacae* complex	ETEST^®^	51 (**100**)	0/3 (**0**)	0/48 (**0**)
	VITEK^®^2	42 (82)	2/3 (67)	7/48 (15)
	Disc	45 (88)	2/3 (67)	4/48 (8)
*Escherichia coli*	ETEST^®^	49 (**100**)	NC	0/49 (**0**)
	VITEK^®^2	46 (**94**)	NC	3/49 (6)
	Disc	43 (88)	NC	6/49 (12)
*Klebsiella aerogenes*	ETEST^®^	45 (**92**)	2/5 (40)	2/44 (5)
	VITEK^®^2	17 (35)	0/5 (**0**)	32/44 (73)
	Disc	44 (**90**)	4/5 (80)	1/44 (2)
*Klebsiella oxytoca*	ETEST^®^	45 (**90**)	1/3 (33)	4/47 (9)
	VITEK^®^2	33 (66)	0/3 (**0**)	17/47 (36)
	Disc	46 (**92**)	0/3 (**0**)	4/47 (9)
*Klebsiella pneumoniae*	ETEST^®^	49 (**100**)	0/3 (**0**)	0/46 (**0**)
	VITEK^®^2	34 (69)	1/3 (33)	14/46 (30)
	Disc	44 (**90**)	0/3 (**0**)	5/46 (11)
*Proteus mirabilis*	ETEST^®^	41 (85)	5/13 (39)	2/35 (6)
	VITEK^®^2	20 (40)	0/14 (**0**)	30/36 (83)
	Disc	42 (84)	4/14 (29)	4/36 (11)

NC, not calculable.

^a^Acceptable values are in bold: categorical agreement (CA) ≥90% and ≤3% major errors (ME) and very major errors (VME).

#### Mecillinam testing

The detailed results of the performance study for mecillinam susceptibility testing by disc diffusion are presented in Table S7.

The three routine methods showed great variabilities between the Enterobacterales species as regards susceptible/resistant categorization. ETEST^®^ CA rates were above the 90% threshold for all tested species, except for *P. mirabilis* (Table 4B). ME rates were below 3%, with the exception of *K. aerogenes* (5%), *P. mirabilis* (6%) and *K. oxytoca* (9%). VITEK^®^2 performances were acceptable for *E. coli* and *C. koseri*, except for the VME for *C. koseri* (3/3 resistant strains erroneously categorized) and ME for *E. coli* (6%). Conversely, VITEK^®^2 overestimated mecillinam MICs for *E. cloacae* complex and *Klebsiella* spp. isolates, with particularly high rates of ME (from 15% to 73%). For these species, disc diffusion more efficiently categorized mecillinam-susceptible and -resistant isolates, with 88% to 92% CA rates and ME rates from 2% to 11%.

## Discussion

Antibiotic resistance among uropathogens is a growing concern, especially cephalosporin- and fluoroquinolone-resistant strains.^1,3,4^ Fosfomycin and mecillinam are relatively old oral antibiotics with activity against MDR Enterobacterales.^9,10,18^ Antibiotic failure in case of UTIs has a substantial impact on patients’ quality of life. Therefore, there is a strong clinical need for correct antimicrobial susceptibility testing to help physicians treating patients with UTIs.^3^ The reference method is AD, which is far too complex and time consuming to be considered outside research settings. Fosfomycin and mecillinam susceptibility testing with the VITEK^®^2 automated system has been validated only for *E. coli.* Many laboratories perform disc diffusion or MIC gradient test strips to determine fosfomycin and mecillinam susceptibility for non-*E.coli* Enterobacterales in addition to their routine technique. Therefore, we determined the activity of fosfomycin and mecillinam with both reference and routine methods (i.e. VITEK^®^2, ETEST^®^ and disc diffusion), against a large collection of *E. coli* and non-*E.coli* Enterobacterales isolated from monomicrobial urines of female patients with microbiological criteria of UTI.^6^

Great discrepancies for fosfomycin susceptibility were observed among the Enterobacterales species. As previously reported, resistant *E. coli* or *C. koseri* isolates were rare.^4,5^ Conversely, 30% to 40% of *E. cloacae* complex and *Klebsiella* spp. isolates were fosfomycin resistant in our study. For these latter species, the three routine techniques overestimated MICs and fosfomycin resistance. The particularly low EA rates for VITEK^®^2 for these species (from 41% for *E. cloacae* complex to 78% for *K. aerogenes*) and the trend to overestimate fosfomycin MICs could be explained by the fosfomycin heteroresistance phenomenon combined with technical discrepancies between AD and the broth microdilution method, which is the base of automated susceptibility tests.^19^ Indeed, the starting inoculum is 2- to 8-fold higher in the broth microdilution method than in AD according to CLSI guidelines. Thus, Ballestero-Téllez *et al.*^19^ showed that the initial inoculum in the microdilution method is enriched with fosfomycin-resistant subpopulations compared with AD, which could partially explain the fosfomycin MIC discrepancies. Of note, a trend to predict higher fosfomycin MICs for *K. pneumoniae* had already been observed for ETEST^®^.^15^

As regards the poor performance for *E. cloacae* complex and *Klebsiella* spp. in terms of CA, VME and ME, they are probably linked with their fosfomycin MIC distributions, with an MIC50 equal to the *E. coli* EUCAST breakpoint of 8 mg/L, and nearly half of the isolates (45% to 55%) with an MIC within one dilution of this breakpoint. Consequently, in our study, fosfomycin could not be considered an interesting therapeutic option for UTI caused by *E. cloacae* complex and *Klebsiella* spp., which accords with the conclusion of the EUCAST general consultation on oral fosfomycin breakpoints for Enterobacterales including microbiological, pharmacokinetic/pharmacodynamic and clinical data: ‘no useful activity’ of oral fosfomycin for *K. pneumoniae*, *K. aerogenes* and *E. cloacae* complex.^20^

In this EUCAST consultation, nothing is mentioned about fosfomycin activity against *P. mirabilis* or *C. koseri* UTIs, probably due to lack of data. Still, in our study, most isolates of these species were susceptible to fosfomycin using the *E. coli* EUCAST breakpoint of 8 mg/L. Moreover, VITEK^®^2 and ETEST^®^ were close to reaching ISO requirements, and CA and ME rates were acceptable for disc diffusion. Further study is needed, with a collection comprising more fosfomycin-resistant strains, to evaluate more precisely VME and bias. Indeed, there were only a few resistant *C. koseri* (*n* = 3) and *E. coli* (*n* = 2) strains in our study, and their MICs (16 mg/L) were just above the 8 mg/L breakpoint.

In 2021, the EUCAST breakpoint for oral fosfomycin changed from 32 to 8 mg/L.^21^ Previous studies comparing several testing methods with AD for non-*E. coli* Enterobacterales (mostly *K. pneumoniae* isolates) used a breakpoint of 32 mg/L^10^ or even 64 mg/L,^22^ making difficult a comparison with our results. Unfortunately, the currently available MIC range for VITEK^®^2 (N372 card, MICs from 16 to 128 mg/L) does not include the 8 mg/L breakpoint. In our study, isolates with a fosfomycin MIC ≤16 mg/L with VITEK^®^2 were considered susceptible, leading to misclassification as susceptible the few *E. coli* and *C. koseri* isolates that were resistant in AD with fosfomycin MIC = 16 mg/L. Of note, the proportion of misclassified strains by VITEK^®^2 among *E. coli* and *C. koseri* isolates from urines of female patients with microbiological criteria of UTI was very low, at 4% (*n* = 2) and 6% (*n* = 3), respectively. However, the development of a novel antimicrobial susceptibility card that could reach the fosfomycin EUCAST breakpoint may improve VITEK^®^2 performances for fosfomycin testing.

Mecillinam was highly active against the tested strains (more than 90% of susceptible strains), apart from *P. mirabilis* isolates (28% of resistant strains), in line with a recent French study.^4^ The three routine methods showed poor performances for *P. mirabilis,* with low EA and CA rates. With the exception of *P. mirabilis*, ETEST^®^ nearly fulfilled the ISO requirements for every species, and CA rates were acceptable for disc diffusion. ISO criteria were reached for *C. koseri* and *E. coli* testing with VITEK^®^2, apart from too high VME rates. The three routine techniques share the same issue of too high VME rates. This common limitation is probably linked with the very low number of mecillinam-resistant isolates in our study: only 31 mecillinam-resistant strains were included, among which 14 were *P. mirabilis.* In comparison to previous European^7^ or French works,^4^ the isolates of our collection were globally more susceptible to the different antibiotic classes. For instance, 34% of our isolates were resistant to ampicillin and 11% were resistant to ofloxacin, whereas the resistance rates reached 50% for ampicillin and 15%–20% for fluoroquinolones in two recent studies performed on urine isolates irrespective of sex or specimen type (midstream, catheter).^4,7^ For our study, we chose to select isolates from monomicrobial midstream urine samples collected from women with significant bacteriuria and leukocyturia (without any urinary catheter), in order to try to exclude nosocomial isolates for which fosfomycin and mecillinam are not first-line treatment options. Further performance study is warranted with a collection of mecillinam-resistant non-*E. coli* Enterobacterales. Fuchs *et al.*^18^ analysed the performance of disc diffusion and MIC gradient test strips for mecillinam susceptibility testing with a collection that was enriched with resistant isolates (78.1%). The VME rate was not acceptable either for disc diffusion or MIC gradient test strips, at 8.5% and 12.2%, respectively. However, mecillinam discs and MIC test strips were not the same as those we used (Oxoid versus Mast for discs, and Liofilchem versus bioMérieux for MIC test strips).

For organizational reasons, two different inoculum suspensions for each strain had to be used for AD and routine methods in our study. Although several controls were set up to minimize the risk that this affected the results (frequent testing with control strains for all methods, double checking in case of significant discrepancy), we cannot exclude that it might explain some of the observed divergences.

In conclusion, there is a strong clinical need for correct fosfomycin and mecillinam susceptibility testing for UTI treatment. The AD reference method is not compatible with routine laboratory diagnostics. Therefore, results of our study show that ETEST^®^ and disc diffusion could be alternatives to AD to determine fosfomycin susceptibility for *C. koseri* and *P. mirabilis* and to determine mecillinam susceptibility for *C. koseri*, *E. cloacae* complex and *Klebsiella* spp. The VITEK^®^2 automated system may also be of interest to assess fosfomycin MICs for *C. koseri* and *P. mirabilis,* and mecillinam MICs for *C. koseri.* Additional studies comprising more fosfomycin- and mecillinam-resistant isolates are needed to strengthen these conclusions.

Our study demonstrates that oral fosfomycin may not be a pertinent first-line antibiotic against UTIs caused by *Klebsiella* spp. and *E. cloacae* complex, because of poor performances of routine methods (VITEK^®^2, ETEST^®^ and disc diffusion) and because of epidemiological data in line with EUCAST conclusions.^20^ Likewise, mecillinam may not be the antibiotic of choice against *P. mirabilis* isolates of our collection.

## Supplementary Material

dkae271_Supplementary_Data

## Supplementary data

Figure S1 and Tables S1 to S7 are available as Supplementary data at *JAC* Online.
