# Supplementary material for: Evaluation of several routine methods for fosfomycin and mecillinam susceptibility testing of Enterobacterales urine isolates
Source: J Antimicrob Chemother. 2024 Aug 19;79(10):2645–52. doi: 10.1093/jac/dkae271 (PMC11441990; doi:10.1093/jac/dkae271)
Supplement: dkae271_Supplementary_Data [file dkae271_supplementary_data.docx]

**B.**

**A.**

**C.**

**E.**

**D.**

**F.**

**G.**

**Figure S1.** Fosfomycin (black) and mecillinam (white) MIC distributions of tested *Citrobacter koseri* (A.), *Enterobacter cloacae* complex (B.), *Escherichia coli* (C.), *Klebsiella aerogenes* (D.), *Klebsiella oxytoca* (E.), *Klebsiella pneumoniae* (F.), and *Proteus mirabilis* (G.) strains with agar dilution reference method.

The vertical dashed lines designate the breakpoints used.

**Table S1.** Rates of acquired non-susceptibility to various antibiotics for the 347 Enterobacterales urine isolates

|  | **Ampicillin** | **Amoxicillin clavulanate** | **3rd generation cephalosporins** | **Ertapenem** | **Cotrimo-xazole** | **Nitro-furantoine** | **Ofloxacin** |
| --- | --- | --- | --- | --- | --- | --- | --- |
| ***Citrobacter koseri*** | - | 0% | 2% | 0% | 0% | 0% | 2% |
| ***Enterobacter cloacae* complex** | - | - | 45% | 10% | 25% | 0% | 27% |
| ***Escherichia coli*** | 22% | 8% | 0% | 0% | 16% | 0% | 6% |
| ***Klebsiella aerogenes*** | - | - | 14% | 0% | 0% | 4% | 0% |
| ***Klebsiella oxytoca*** | - | 16% | 10% | 2% | 4% | 0% | 6% |
| ***Klebsiella pneumoniae*** | - | 14% | 4% | 0% | 10% | 8% | 8% |
| ***Proteus mirabilis*** | 46% | 26% | 2% | 0% | 32% | - | 26% |
| **Total** | 34% | 13% | 11% | 2% | 13% | 2% | 11% |

**Table S2.** Fosfomycin agar dilution MIC against Etest^®^ MIC for *Citrobacter koseri* (A.), *Enterobacter cloacae* complex (B.), *Escherichia coli* (C.), *Klebsiella aerogenes* (D.), *Klebsiella oxytoca* (E.), *Klebsiella pneumoniae* (F.), and *Proteus mirabilis* (G.) strains.

The black lines designate the breakpoint used. Cells with essential agreement are shaded in gray

**A.**

|  | **Agar dilution MIC (mg/L)** | | | | | | |
| --- | --- | --- | --- | --- | --- | --- | --- |
| **Etest MIC (mg/L)** | **0.25** | **0.5** | **1** | **2** | **4** | **8** | **16** |
| **0.25** | 4 | 3 | 1 |  |  |  |  |
| **0.5** | 2 | 8 | 1 |  |  |  | 1 |
| **1** |  | 16 | 3 | 1 |  |  |  |
| **2** |  |  | 5 |  |  |  |  |
| **4** |  |  |  | 2 |  |  |  |
| **8** |  |  |  |  |  |  | 1 |
| **16** |  |  |  |  |  |  | 1 |

**B.**

|  | **Agar dilution MIC (mg/L)** | | | | | | | | | | | |
| --- | --- | --- | --- | --- | --- | --- | --- | --- | --- | --- | --- | --- |
| **Etest MIC (mg/L)** | **0.25** | **0.5** | **1** | **2** | **4** | **8** | **16** | **32** | **64** | **128** | **256** | **≥ 512** |
| **0.25** |  | 1 |  |  | 1 |  |  |  |  |  |  |  |
| **0.5** |  | 1 |  |  |  |  |  |  |  |  |  |  |
| **1** |  |  | 2 |  |  |  |  |  |  |  |  |  |
| **2** |  |  | 2 | 1 |  |  |  |  |  |  |  |  |
| **4** |  |  |  |  | 4 |  |  |  |  |  |  |  |
| **8** |  |  |  |  | 4 | 8 |  | 2 |  |  |  |  |
| **16** |  |  |  |  |  | 6 | 6 | 1 |  |  |  |  |
| **32** |  |  |  |  |  |  | 3 | 3 |  |  |  |  |
| **64** |  |  |  |  |  |  |  | 1 |  |  |  |  |
| **128** |  |  |  |  |  |  |  |  | 2 |  |  |  |
| **256** |  |  |  |  |  |  |  |  |  |  |  |  |
| **≥ 512** |  |  |  |  |  |  |  |  |  | 1 |  | 2 |

**C.**

|  | **Agar dilution MIC (mg/L)** | | | | | | |
| --- | --- | --- | --- | --- | --- | --- | --- |
| **Etest MIC (mg/L)** | **0.25** | **0.5** | **1** | **2** | **4** | **8** | **16** |
| **0.25** | 4 | 8 |  |  |  |  |  |
| **0.5** | 1 | 17 | 4 |  |  |  |  |
| **1** |  | 2 | 4 | 4 |  |  |  |
| **2** |  |  | 2 |  |  |  |  |
| **4** |  |  |  |  |  |  |  |
| **8** |  |  |  |  | 1 |  | 1 |
| **16** |  |  |  |  |  |  | 1 |

**D.**

|  | **Agar dilution MIC (mg/L)** | | | | | | | | | | |
| --- | --- | --- | --- | --- | --- | --- | --- | --- | --- | --- | --- |
| **Etest MIC (mg/L)** | **0.25** | **0.5** | **1** | **2** | **4** | **8** | **16** | **32** | **64** | **128** | **256** |
| **0.25** |  |  |  |  | 1 |  |  |  |  |  |  |
| **0.5** |  |  |  |  |  |  |  |  |  |  |  |
| **1** |  |  |  |  |  |  |  |  |  |  |  |
| **2** |  |  |  | 1 |  |  |  |  |  |  |  |
| **4** |  |  |  | 5 | 1 |  |  |  |  |  |  |
| **8** |  |  |  |  | 12 | 7 |  | 1 |  |  |  |
| **16** |  |  |  |  | 1 | 7 | 3 |  |  |  |  |
| **32** |  |  |  |  |  |  | 5 | 3 |  |  |  |
| **64** |  |  |  |  |  |  |  | 1 |  |  |  |
| **128** |  |  |  |  |  |  |  |  |  |  |  |
| **256** |  |  |  |  |  |  |  |  |  |  |  |
| **≥ 512** |  |  |  |  |  |  |  |  |  |  | 1 |

**E.**

|  | **Agar dilution MIC (mg/L)** | | | | | | | | | | |
| --- | --- | --- | --- | --- | --- | --- | --- | --- | --- | --- | --- |
| **Etest MIC (mg/L)** | **0.5** | **1** | **2** | **4** | **8** | **16** | **32** | **64** | **128** | **256** | **≥ 512** |
| **0.5** |  | 1 |  |  |  |  |  |  |  |  |  |
| **1** |  |  |  |  |  |  |  |  |  |  |  |
| **2** |  | 1 |  |  |  |  |  |  |  |  |  |
| **4** |  |  | 4 | 4 | 1 |  |  |  |  |  |  |
| **8** |  |  |  | 8 | 7 | 1 |  |  |  |  |  |
| **16** |  |  |  |  | 6 | 3 | 1 |  |  |  |  |
| **32** |  |  |  |  |  | 6 | 1 |  |  |  |  |
| **64** |  |  |  |  |  |  | 5 |  |  |  |  |
| **128** |  |  |  |  |  |  |  |  |  |  |  |
| **256** |  |  |  |  |  |  |  |  |  |  |  |
| **≥ 512** |  |  |  |  |  |  |  |  |  |  | 1 |

**F.**

|  | **Agar dilution MIC (mg/L)** | | | | | | | | | | |
| --- | --- | --- | --- | --- | --- | --- | --- | --- | --- | --- | --- |
| **Etest MIC (mg/L)** | **0.5** | **1** | **2** | **4** | **8** | **16** | **32** | **64** | **128** | **256** | **≥ 512** |
| **0.5** |  | 1 |  |  |  |  |  |  |  |  |  |
| **1** |  |  | 1 |  |  |  |  |  |  |  |  |
| **2** |  |  | 2 | 1 |  |  |  |  |  |  |  |
| **4** |  |  |  | 2 |  |  |  |  |  |  |  |
| **8** |  |  |  | 9 | 7 | 1 |  |  |  |  |  |
| **16** |  |  |  |  | 13 | 1 | 1 |  |  |  |  |
| **32** |  |  |  |  |  | 5 | 1 |  |  |  |  |
| **64** |  |  |  |  |  |  | 1 |  |  |  |  |
| **128** |  |  |  |  |  |  |  |  |  | 1 |  |
| **256** |  |  |  |  |  |  |  |  |  |  |  |
| **≥ 512** |  |  |  |  |  |  |  |  |  | 1 | 1 |

**G.**

|  | **Agar dilution MIC (mg/L)** | | | | | | | | | | | |
| --- | --- | --- | --- | --- | --- | --- | --- | --- | --- | --- | --- | --- |
| **Etest MIC (mg/L)** | **0.25** | **0.5** | **1** | **2** | **4** | **8** | **16** | **32** | **64** | **128** | **256** | **≥ 512** |
| **0.25** | 3 | 1 | 1 |  |  |  |  |  |  |  |  |  |
| **0.5** | 1 | 3 | 2 | 2 |  | 1 |  |  |  |  |  |  |
| **1** |  | 8 | 4 | 2 |  |  |  |  |  |  |  |  |
| **2** |  |  | 7 | 1 | 1 |  |  |  |  |  |  |  |
| **4** |  |  |  |  | 1 |  |  |  |  |  |  |  |
| **8** |  |  | 1 |  |  |  |  |  |  |  |  |  |
| **16** |  |  |  |  |  |  | 1 |  |  |  |  |  |
| **32** |  |  |  |  |  |  |  |  | 1 |  |  |  |
| **64** |  |  |  |  |  |  |  |  | 2 |  |  |  |
| **128** |  |  |  |  |  |  |  |  |  | 1 |  |  |
| **256** |  |  |  |  |  |  |  |  |  |  |  |  |
| **≥ 512** |  |  |  |  |  |  |  |  |  | 1 | 1 | 4 |

**Table S3.** Fosfomycin agar dilution MIC against Vitek^®^2 MIC for *Citrobacter koseri* (A.), *Enterobacter cloacae* complex (B.), *Escherichia coli* (C.), *Klebsiella aerogenes* (D.), *Klebsiella oxytoca* (E.), *Klebsiella pneumoniae* (F.), and *Proteus mirabilis* (G.) strains.

The black lines designate the breakpoint used. Cells with essential agreement are shaded in gray

**A.**

|  | **Agar dilution MIC (mg/L)** | | | | | | |
| --- | --- | --- | --- | --- | --- | --- | --- |
| **Vitek**^®^**2 MIC (mg/L)** | **0.25** | **0.5** | **1** | **2** | **4** | **8** | **16** |
| **≤ 16** | 6 | 27 | 10 | 3 |  |  | 3 |
| **32** |  |  |  |  |  |  |  |

**B.**

|  | **Agar dilution MIC (mg/L)** | | | | | | | | | | |
| --- | --- | --- | --- | --- | --- | --- | --- | --- | --- | --- | --- |
| **Vitek**^®^**2 MIC (mg/L)** | **0.5** | **1** | **2** | **4** | **8** | **16** | **32** | **64** | **128** | **256** | **≥ 512** |
| **≤ 16** | 2 | 4 | 1 | 6 | 2 |  |  |  |  |  |  |
| **32** |  |  |  | 3 | 6 | 1 | 2 |  |  |  |  |
| **64** |  |  |  |  | 5 | 3 |  |  |  |  |  |
| **128** |  |  |  |  | 1 | 5 | 1 |  | 1 |  |  |
| **≥ 256** |  |  |  |  |  |  | 4 | 2 |  |  | 2 |

**C.**

|  | **Agar dilution MIC (mg/L)** | | | | | | |
| --- | --- | --- | --- | --- | --- | --- | --- |
| **Vitek**^®^**2 MIC (mg/L)** | **0.25** | **0.5** | **1** | **2** | **4** | **8** | **16** |
| **≤ 16** | 5 | 27 | 10 | 4 | 1 |  | 2 |
| **32** |  |  |  |  |  |  |  |

**D.**

|  | **Agar dilution MIC (mg/L)** | | | | | | | |
| --- | --- | --- | --- | --- | --- | --- | --- | --- |
| **Vitek**^®^**2 MIC (mg/L)** | **2** | **4** | **8** | **16** | **32** | **64** | **128** | **256** |
| **≤ 16** | 6 | 14 | 12 | 1 | 1 |  |  |  |
| **32** |  |  | 1 | 1 | 2 |  |  |  |
| **64** |  | 1 | 1 | 4 |  |  |  |  |
| **128** |  |  |  | 2 |  |  |  |  |
| **≥ 256** |  |  |  |  | 2 |  |  | 1 |

**E.**

|  | **Agar dilution MIC (mg/L)** | | | | | | | | | |
| --- | --- | --- | --- | --- | --- | --- | --- | --- | --- | --- |
| **Vitek**^®^**2 MIC (mg/L)** | **1** | **2** | **4** | **8** | **16** | **32** | **64** | **128** | **256** | **≥ 512** |
| **≤ 16** | 2 | 4 | 9 | 4 | 1 | 1 |  |  |  |  |
| **32** |  |  | 3 | 2 | 3 |  |  |  |  |  |
| **64** |  |  |  | 6 | 1 |  |  |  |  |  |
| **128** |  |  |  | 2 | 3 | 3 |  |  |  |  |
| **≥ 256** |  |  |  |  | 2 | 3 |  |  |  | 1 |

**F.**

|  | **Agar dilution MIC (mg/L)** | | | | | | | | | |
| --- | --- | --- | --- | --- | --- | --- | --- | --- | --- | --- |
| **Vitek**^®^**2 MIC (mg/L)** | **1** | **2** | **4** | **8** | **16** | **32** | **64** | **128** | **256** | **≥ 512** |
| **≤ 16** | 1 | 3 | 11 | 11 | 1 |  |  |  |  |  |
| **32** |  |  | 1 | 4 |  | 2 |  |  |  |  |
| **64** |  |  |  | 5 | 4 |  |  |  |  |  |
| **128** |  |  |  |  | 2 |  |  |  |  |  |
| **≥ 256** |  |  |  |  |  | 1 |  |  | 2 | 1 |

**G.**

|  | **Agar dilution MIC (mg/L)** | | | | | | | | | | | |
| --- | --- | --- | --- | --- | --- | --- | --- | --- | --- | --- | --- | --- |
| **Vitek**^®^**2 MIC (mg/L)** | **0.25** | **0.5** | **1** | **2** | **4** | **8** | **16** | **32** | **64** | **128** | **256** | **≥ 512** |
| **≤ 16** | 4 | 12 | 15 | 5 | 2 | 1 | 1 |  |  | 1 |  |  |
| **32** |  |  |  |  |  |  |  |  | 1 |  | 1 |  |
| **64** |  |  |  |  |  |  |  |  | 1 |  |  |  |
| **128** |  |  |  |  |  |  |  |  |  |  |  |  |
| **≥ 256** |  |  |  |  |  |  |  |  | 1 | 1 |  | 4 |

**Table S4.** Mecillinam agar dilution MIC against Etest^®^ MIC for *Citrobacter koseri* (A.), *Enterobacter cloacae* complex (B.), *Escherichia coli* (C.), *Klebsiella aerogenes* (D.), *Klebsiella oxytoca* (E.), *Klebsiella pneumoniae* (F.), and *Proteus mirabilis* (G.) strains.

The black lines designate the breakpoint used. Cells with essential agreement are shaded in gray

**A.**

|  | **Agar dilution (mg/L)** | | | | | | | | | | | | |
| --- | --- | --- | --- | --- | --- | --- | --- | --- | --- | --- | --- | --- | --- |
| **Etest MIC (mg/L)** | **0.06** | **0.12** | **0.25** | **0.5** | **1** | **2** | **4** | **8** | **16** | **32** | **64** | **128** | **≥ 256** |
| **0.06** | 3 | 4 |  | 1 |  |  |  |  |  |  |  |  |  |
| **0.12** | 4 | 19 | 3 |  |  |  |  |  |  |  |  |  |  |
| **0.25** |  | 2 | 3 | 1 |  |  |  |  |  |  |  |  |  |
| **0.5** |  |  | 5 | 1 |  |  |  |  |  |  |  |  |  |
| **1** |  |  |  |  |  |  |  |  |  |  |  |  |  |
| **2** |  |  |  |  |  |  |  |  |  |  |  |  |  |
| **4** |  |  |  |  |  |  |  |  |  |  |  |  |  |
| **8** |  |  |  |  |  |  |  |  |  |  |  |  |  |
| **16** |  |  |  |  |  |  |  |  |  |  |  |  |  |
| **32** |  |  |  |  |  |  |  |  |  |  |  |  |  |
| **64** |  |  |  |  |  |  |  |  |  |  |  |  |  |
| **128** |  |  |  |  |  |  |  |  |  |  |  |  |  |
| **≥ 256** |  |  |  |  |  |  |  |  |  |  |  |  | 3 |

**B.**

|  | **Agar dilution (mg/L)** | | | | | | | | | | | |
| --- | --- | --- | --- | --- | --- | --- | --- | --- | --- | --- | --- | --- |
| **Etest MIC (mg/L)** | **0.12** | **0.25** | **0.5** | **1** | **2** | **4** | **8** | **16** | **32** | **64** | **128** | **≥ 256** |
| **0.12** | 2 | 2 |  |  |  |  |  |  |  |  |  |  |
| **0.25** | 2 | 11 | 6 |  |  |  |  |  |  |  |  |  |
| **0.5** |  | 1 | 7 | 3 |  |  |  |  |  |  |  |  |
| **1** |  |  |  | 6 | 1 |  |  |  |  |  |  |  |
| **2** |  |  |  | 2 | 1 |  |  |  |  |  |  |  |
| **4** |  |  |  |  |  | 1 |  |  |  |  |  |  |
| **8** |  |  |  |  |  |  | 3 |  |  |  |  |  |
| **16** |  |  |  |  |  |  |  | 1 |  |  |  |  |
| **32** |  |  |  |  |  |  |  |  |  |  |  |  |
| **64** |  |  |  |  |  |  |  |  |  |  |  |  |
| **128** |  |  |  |  |  |  |  |  |  |  |  |  |
| **≥ 256** |  |  |  |  |  |  |  |  |  | 1 |  | 1 |

**C.**

|  | **Agar dilution (mg/L)** | | | | | | | |
| --- | --- | --- | --- | --- | --- | --- | --- | --- |
| **Etest MIC (mg/L)** | **0.12** | **0.25** | **0.5** | **1** | **2** | **4** | **8** | **16** |
| **0.12** | 10 | 5 |  |  |  |  |  |  |
| **0.25** | 1 | 10 | 1 |  |  |  |  |  |
| **0.5** |  | 3 | 2 |  |  |  |  |  |
| **1** |  |  | 5 | 1 |  |  |  |  |
| **2** |  |  |  | 6 | 2 | 1 |  |  |
| **4** |  |  |  |  | 1 | 1 |  |  |
| **8** |  |  |  |  |  |  |  |  |
| **16** |  |  |  |  |  |  |  |  |

**D.**

|  | **Agar dilution (mg/L)** | | | | | | | | | | | |
| --- | --- | --- | --- | --- | --- | --- | --- | --- | --- | --- | --- | --- |
| **Etest MIC (mg/L)** | **0.12** | **0.25** | **0.5** | **1** | **2** | **4** | **8** | **16** | **32** | **64** | **128** | **≥ 256** |
| **0.12** |  | 1 |  |  |  |  |  |  |  |  |  |  |
| **0.25** |  |  | 15 |  |  |  |  |  |  |  |  |  |
| **0.5** |  |  | 5 | 11 |  |  |  |  | 1 |  |  |  |
| **1** |  |  | 4 |  |  |  |  | 1 |  |  |  |  |
| **2** |  |  | 3 |  | 1 |  |  |  |  |  |  |  |
| **4** |  |  |  |  | 1 |  |  |  |  |  |  |  |
| **8** |  |  |  |  |  |  | 1 |  |  |  |  |  |
| **16** |  |  |  |  |  |  |  |  |  |  |  |  |
| **32** |  |  |  |  |  |  |  |  |  |  |  |  |
| **64** |  |  |  |  |  |  |  |  |  |  |  |  |
| **128** |  |  |  | 1 |  |  |  |  |  |  |  |  |
| **≥ 256** |  |  |  | 1 |  |  |  |  |  | 2 |  | 1 |

**E.**

|  | **Agar dilution (mg/L)** | | | | | | | | | | |
| --- | --- | --- | --- | --- | --- | --- | --- | --- | --- | --- | --- |
| **Etest MIC (mg/L)** | **0.12** | **0.25** | **0.5** | **1** | **2** | **4** | **8** | **16** | **32** | **64** | **128** |
| **0.12** |  | 2 |  |  |  |  |  |  |  |  |  |
| **0.25** |  | 10 | 12 |  |  |  |  |  |  |  |  |
| **0.5** |  | 3 | 5 | 1 |  |  |  |  |  |  |  |
| **1** |  |  | 2 |  |  |  |  |  |  |  |  |
| **2** |  |  |  | 1 | 1 | 3 |  |  |  |  |  |
| **4** |  | 1 |  |  | 2 |  |  |  |  |  |  |
| **8** |  |  |  |  |  |  |  | 1 |  |  |  |
| **16** |  |  |  |  |  |  |  |  |  |  |  |
| **32** |  |  |  |  |  |  |  |  |  |  |  |
| **64** |  | 1 |  |  |  |  |  |  |  |  |  |
| **128** |  |  |  |  |  |  |  |  |  |  |  |
| **≥ 256** |  | 1 |  |  | 1 | 1 |  | 1 |  |  | 1 |

**F.**

|  | **Agar dilution (mg/L)** | | | | | | | | | | | |
| --- | --- | --- | --- | --- | --- | --- | --- | --- | --- | --- | --- | --- |
| **Etest MIC (mg/L)** | **0.12** | **0.25** | **0.5** | **1** | **2** | **4** | **8** | **16** | **32** | **64** | **128** | **≥ 256** |
| **0.12** | 2 | 9 |  |  |  |  |  |  |  |  |  |  |
| **0.25** | 1 | 11 | 2 |  |  |  |  |  |  |  |  |  |
| **0.5** |  | 4 | 3 | 1 |  |  |  |  |  |  |  |  |
| **1** |  |  | 4 | 2 |  |  |  |  |  |  |  |  |
| **2** |  |  |  | 2 |  | 1 | 1 |  |  |  |  |  |
| **4** |  |  |  |  | 1 | 1 |  |  |  |  |  |  |
| **8** |  |  |  |  |  |  | 1 |  |  |  |  |  |
| **16** |  |  |  |  |  |  |  | 1 |  |  |  |  |
| **32** |  |  |  |  |  |  |  | 1 |  |  |  |  |
| **64** |  |  |  |  |  |  |  |  |  |  |  |  |
| **128** |  |  |  |  |  |  |  |  |  |  |  |  |
| **≥ 256** |  |  |  |  |  |  |  |  |  |  |  | 1 |

**G.**

|  | **Agar dilution (mg/L)** | | | | | | | | | | | |
| --- | --- | --- | --- | --- | --- | --- | --- | --- | --- | --- | --- | --- |
| **Etest MIC (mg/L)** | **0.12** | **0.25** | **0.5** | **1** | **2** | **4** | **8** | **16** | **32** | **64** | **128** | **≥ 256** |
| **0.12** | 1 | 1 |  |  |  |  |  |  |  |  |  |  |
| **0.25** |  | 3 | 2 |  |  |  |  |  |  |  |  |  |
| **0.5** |  | 1 | 2 | 2 |  | 2 | 1 |  |  |  |  |  |
| **1** | 1 |  | 5 | 2 | 5 | 1 |  |  |  |  |  | 1 |
| **2** |  |  |  | 1 |  |  | 1 |  |  |  |  | 2 |
| **4** |  |  |  |  |  |  |  | 1 | 1 |  |  |  |
| **8** |  |  |  |  |  | 2 |  |  |  |  |  |  |
| **16** |  |  |  |  |  |  |  |  |  |  |  |  |
| **32** |  |  |  |  |  |  |  |  |  |  |  |  |
| **64** |  |  |  |  |  |  |  |  |  |  |  |  |
| **128** |  |  |  |  |  |  |  |  |  |  |  |  |
| **≥ 256** |  |  |  |  |  | 1 | 1 |  | 2 |  | 2 | 4 |

**Table S5.** Mecillinam agar dilution MIC against Vitek^®^2 MIC for *Citrobacter koseri* (A.), *Enterobacter cloacae* complex (B.), *Escherichia coli* (C.), *Klebsiella aerogenes* (D.), *Klebsiella oxytoca* (E.), *Klebsiella pneumoniae* (F.), and *Proteus mirabilis* (G.) strains.

The black lines designate the breakpoint used. Cells with essential agreement are shaded in gray

**A.**

|  | **Agar dilution (mg/L)** | | | | | | | | | | | | |
| --- | --- | --- | --- | --- | --- | --- | --- | --- | --- | --- | --- | --- | --- |
| **Vitek**^®^**2 MIC (mg/L)** | **0.06** | **0.12** | **0.25** | **0.5** | **1** | **2** | **4** | **8** | **16** | **32** | **64** | **128** | **≥ 256** |
| **≤ 1** | 7 | 25 | 11 | 3 |  |  |  |  |  |  |  |  | 1 |
| **2** |  |  |  |  |  |  |  |  |  |  |  |  |  |
| **4** |  |  |  |  |  |  |  |  |  |  |  |  | 2 |
| **8** |  |  |  |  |  |  |  |  |  |  |  |  |  |
| **16** |  |  |  |  |  |  |  |  |  |  |  |  |  |

**B.**

|  | **Agar dilution (mg/L)** | | | | | | | | | | | | |
| --- | --- | --- | --- | --- | --- | --- | --- | --- | --- | --- | --- | --- | --- |
| **Vitek**^®^**2 MIC (mg/L)** | **0.06** | **0.12** | **0.25** | **0.5** | **1** | **2** | **4** | **8** | **16** | **32** | **64** | **128** | **≥ 256** |
| **≤ 1** |  | 4 | 13 | 8 | 4 |  |  |  |  |  | 1 |  |  |
| **2** |  |  |  | 1 | 4 |  |  |  |  |  |  |  | 1 |
| **4** |  |  |  | 3 | 2 | 1 |  |  |  |  |  |  |  |
| **8** |  |  |  |  |  | 1 |  |  |  |  |  |  |  |
| **16** |  |  |  |  |  |  | 1 | 2 |  |  |  |  |  |
| **32** |  |  |  |  |  |  |  |  |  |  |  |  |  |
| **≥ 64** |  |  | 1 | 1 | 1 |  |  | 1 | 1 |  |  |  |  |

**C.**

|  | **Agar dilution (mg/L)** | | | | | | | |
| --- | --- | --- | --- | --- | --- | --- | --- | --- |
| **Vitek**^®^**2 MIC (mg/L)** | **0.12** | **0.25** | **0.5** | **1** | **2** | **4** | **8** | **16** |
| **≤ 1** | 11 | 18 | 8 | 4 | 1 |  |  |  |
| **2** |  |  |  | 3 | 1 |  |  |  |
| **4** |  |  |  |  |  |  |  |  |
| **8** |  |  |  |  |  |  |  |  |
| **16** |  |  |  |  | 1 | 2 |  |  |
| **32** |  |  |  |  |  |  |  |  |

**D.**

|  | **Agar dilution (mg/L)** | | | | | | | | | | |
| --- | --- | --- | --- | --- | --- | --- | --- | --- | --- | --- | --- |
| **Vitek**^®^**2 MIC (mg/L)** | **0.25** | **0.5** | **1** | **2** | **4** | **8** | **16** | **32** | **64** | **128** | **≥ 256** |
| **≤ 1** | 1 | 5 | 1 |  |  |  |  |  |  |  |  |
| **2** |  |  |  |  |  |  |  |  |  |  |  |
| **4** |  | 2 | 2 | 1 |  |  |  |  |  |  |  |
| **8** |  |  |  |  |  |  |  |  |  |  |  |
| **16** |  |  |  |  |  |  |  |  |  |  |  |
| **32** |  |  |  |  |  |  |  |  |  |  |  |
| **≥ 64** |  | 20 | 10 | 1 |  | 1 | 1 | 1 | 2 |  | 1 |

**E.**

|  | **Agar dilution (mg/L)** | | | | | | | | | |
| --- | --- | --- | --- | --- | --- | --- | --- | --- | --- | --- |
| **Vitek**^®^**2 MIC (mg/L)** | **0.25** | **0.5** | **1** | **2** | **4** | **8** | **16** | **32** | **64** | **128** |
| **≤ 1** | 11 | 7 | 1 |  |  |  |  |  |  |  |
| **2** |  |  |  |  |  |  |  |  |  |  |
| **4** | 2 | 7 |  | 1 |  |  |  |  |  |  |
| **8** |  |  |  | 1 |  |  |  |  |  |  |
| **16** | 1 |  |  | 1 |  |  |  |  |  |  |
| **32** |  |  |  |  |  |  |  |  |  |  |
| **≥ 64** | 4 | 5 | 1 | 1 | 4 |  | 2 |  |  | 1 |

**F.**

|  | **Agar dilution (mg/L)** | | | | | | | | | | | | |
| --- | --- | --- | --- | --- | --- | --- | --- | --- | --- | --- | --- | --- | --- |
| **Vitek**^®^**2 MIC (mg/L)** | **0.06** | **0.12** | **0.25** | **0.5** | **1** | **2** | **4** | **8** | **16** | **32** | **64** | **128** | **≥ 256** |
| **≤ 1** |  | 3 | 18 | 2 |  |  |  |  |  |  |  |  |  |
| **2** |  |  |  |  | 1 |  |  |  |  |  |  |  |  |
| **4** |  |  | 3 | 3 | 2 |  |  |  |  |  |  |  | 1 |
| **8** |  |  |  |  |  |  |  |  |  |  |  |  |  |
| **16** |  |  |  |  |  |  |  |  | 1 |  |  |  |  |
| **32** |  |  |  |  |  |  |  |  |  |  |  |  |  |
| **≥ 64** |  |  | 3 | 4 | 2 | 1 | 2 | 2 | 1 |  |  |  |  |

**G.**

|  | **Agar dilution (mg/L)** | | | | | | | | | | | | |
| --- | --- | --- | --- | --- | --- | --- | --- | --- | --- | --- | --- | --- | --- |
| **Vitek**^®^**2 MIC (mg/L)** | **0.06** | **0.12** | **0.25** | **0.5** | **1** | **2** | **4** | **8** | **16** | **32** | **64** | **128** | **≥ 256** |
| **≤ 1** |  | 1 | 2 | 1 | 1 | 1 |  |  |  |  |  |  |  |
| **2** |  |  |  |  |  |  |  |  |  |  |  |  |  |
| **4** |  |  |  |  |  |  |  |  |  |  |  |  |  |
| **8** |  |  |  |  |  |  |  |  |  |  |  |  |  |
| **16** |  |  |  |  |  |  |  |  |  |  |  |  |  |
| **32** |  |  |  |  |  |  |  |  |  |  |  |  |  |
| **≥ 64** |  | 1 | 4 | 8 | 4 | 4 | 6 | 3 | 1 | 3 | 1 | 2 | 7 |

**Table S6.** Fosfomycin agar dilution MIC against disc diffusion for *Citrobacter koseri* (A.), *Enterobacter cloacae* complex (B.), *Escherichia coli* (C.), *Klebsiella aerogenes* (D.), *Klebsiella oxytoca* (E.), *Klebsiella pneumoniae* (F.), and *Proteus mirabilis* (G.) strains.

The black lines designate the breakpoint used.

**A.**

|  | **Agar dilution (mg/L)** | | | | | | |
| --- | --- | --- | --- | --- | --- | --- | --- |
| **Diameter (mm)** | **0.25** | **0.50** | **1** | **2** | **4** | **8** | **16** |
| **20** |  |  |  |  |  |  | 1 |
| **21** |  |  |  |  |  |  |  |
| **22** |  |  |  |  |  |  |  |
| **23** |  |  |  |  |  |  |  |
| **24** |  |  | 2 |  |  |  |  |
| **25** |  |  | 1 | 1 |  |  |  |
| **26** |  | 6 | 4 | 2 |  |  | 1 |
| **27** | 1 | 7 |  |  |  |  | 1 |
| **28** | 1 | 3 | 1 |  |  |  |  |
| **29** | 1 | 5 |  |  |  |  |  |
| **30** | 2 | 3 | 1 |  |  |  |  |
| **31** | 1 | 1 |  |  |  |  |  |
| **32** |  | 2 | 1 |  |  |  |  |

**B.**

|  | **Agar dilution (mg/L)** | | | | | | | | | | |
| --- | --- | --- | --- | --- | --- | --- | --- | --- | --- | --- | --- |
| **Diameter (mm)** | **0.5** | **1** | **2** | **4** | **8** | **16** | **32** | **64** | **128** | **256** | **≥ 512** |
| **6** |  |  |  |  |  |  |  |  | 1 |  | 1 |
| **7** |  |  |  |  |  |  |  |  |  |  |  |
| **8** |  |  |  |  |  |  |  | 1 |  |  |  |
| **9** |  |  |  |  |  |  |  |  |  |  |  |
| **10** |  |  |  |  |  |  |  |  |  |  |  |
| **11** |  |  |  |  |  |  |  |  |  |  |  |
| **12** |  |  |  |  |  |  |  |  |  |  |  |
| **13** |  |  |  |  |  |  |  |  |  |  |  |
| **14** |  |  |  |  |  |  |  |  |  |  |  |
| **15** |  |  |  |  |  |  |  |  |  |  |  |
| **16** |  |  |  |  |  |  | 1 |  |  |  |  |
| **17** |  |  |  |  |  | 1 |  |  |  |  |  |
| **18** |  |  |  |  | 1 | 1 | 3 |  |  |  | 1 |
| **19** |  |  |  |  | 3 | 2 | 2 |  |  |  |  |
| **20** |  |  |  | 2 | 3 | 2 |  | 1 |  |  |  |
| **21** |  |  |  | 1 | 2 | 2 | 1 |  |  |  |  |
| **22** |  |  |  | 4 | 3 | 1 |  |  |  |  |  |
| **23** |  |  |  | 1 | 1 |  |  |  |  |  |  |
| **24** |  | 2 |  |  | 1 |  |  |  |  |  |  |
| **25** |  | 1 |  |  |  |  |  |  |  |  |  |
| **26** | 1 | 1 | 1 | 1 |  |  |  |  |  |  |  |
| **27** |  |  |  |  |  |  |  |  |  |  |  |
| **28** |  |  |  |  |  |  |  |  |  |  |  |
| **29** |  |  |  |  |  |  |  |  |  |  |  |
| **30** | 1 |  |  |  |  |  |  |  |  |  |  |

**C.**

|  | **Agar dilution (mg/L)** | | | | | | | |
| --- | --- | --- | --- | --- | --- | --- | --- | --- |
| **Diameter (mm)** | **0.25** | **0.5** | **1** | **2** | **4** | **8** | **16** | **32** |
| **22** |  |  |  |  |  |  |  |  |
| **23** |  |  |  |  |  |  |  |  |
| **24** |  |  |  |  |  |  |  |  |
| **25** | 1 | 1 | 2 | 1 | 1 |  |  |  |
| **26** | 2 | 7 | 2 | 2 |  |  |  |  |
| **27** |  | 2 | 3 | 1 |  |  |  |  |
| **28** |  | 6 | 2 |  |  |  | 1 |  |
| **29** |  | 5 | 1 |  |  |  |  |  |
| **30** |  | 5 |  |  |  |  | 1 |  |
| **31** | 1 | 1 |  |  |  |  |  |  |
| **32** |  |  |  |  |  |  |  |  |
| **33** |  |  |  |  |  |  |  |  |
| **34** |  |  |  |  |  |  |  |  |
| **35** |  |  |  |  |  |  |  |  |
| **36** | 1 |  |  |  |  |  |  |  |

**D.**

|  | **Agar dilution (mg/L)** | | | | | | | |
| --- | --- | --- | --- | --- | --- | --- | --- | --- |
| **Diameter (mm)** | **2** | **4** | **8** | **16** | **32** | **64** | **128** | **256** |
| **6** |  |  |  |  |  |  |  | 1 |
| **7** |  |  |  |  |  |  |  |  |
| **8** |  |  |  |  |  |  |  |  |
| **9** |  |  |  |  |  |  |  |  |
| **10** |  |  | 1 |  | 1 |  |  |  |
| **11** |  |  |  |  |  |  |  |  |
| **12** |  | 1 |  | 1 |  |  |  |  |
| **13** |  |  |  |  |  |  |  |  |
| **14** |  |  |  |  |  |  |  |  |
| **15** |  |  |  |  |  |  |  |  |
| **16** |  |  | 1 |  | 1 |  |  |  |
| **17** |  | 1 | 1 | 1 |  |  |  |  |
| **18** | 1 |  | 3 | 4 |  |  |  |  |
| **19** | 1 | 3 | 5 | 2 |  |  |  |  |
| **20** | 1 | 6 | 3 |  | 2 |  |  |  |
| **21** | 3 | 2 |  |  |  |  |  |  |
| **22** |  | 2 |  |  |  |  |  |  |
| **23** |  |  |  |  |  |  |  |  |
| **24** |  |  |  |  |  |  |  |  |

**E.**

|  | **Agar dilution (mg/L)** | | | | | | | | | |
| --- | --- | --- | --- | --- | --- | --- | --- | --- | --- | --- |
| **Diameter (mm)** | **1** | **2** | **4** | **8** | **16** | **32** | **64** | **128** | **256** | **≥ 512** |
| **14** |  |  |  |  |  | 2 |  |  |  |  |
| **15** |  |  |  | 1 | 2 | 2 |  |  |  | 1 |
| **16** |  |  | 2 |  | 1 | 3 |  |  |  |  |
| **17** |  |  |  | 1 | 2 |  |  |  |  |  |
| **18** |  |  | 1 | 3 | 3 |  |  |  |  |  |
| **19** |  |  | 2 | 3 | 1 |  |  |  |  |  |
| **20** | 1 | 2 | 4 | 4 | 1 |  |  |  |  |  |
| **21** |  | 1 | 2 | 2 |  |  |  |  |  |  |
| **22** |  | 1 |  |  |  |  |  |  |  |  |
| **23** |  |  |  |  |  |  |  |  |  |  |
| **24** |  |  |  |  |  |  |  |  |  |  |
| **25** | 1 |  |  |  |  |  |  |  |  |  |
| **26** |  |  | 1 |  |  |  |  |  |  |  |

**F.**

|  | **Agar dilution (mg/L)** | | | | | | | | | |
| --- | --- | --- | --- | --- | --- | --- | --- | --- | --- | --- |
| **Diameter (mm)** | **1** | **2** | **4** | **8** | **16** | **32** | **64** | **128** | **256** | **≥ 512** |
| **6** |  |  |  |  |  |  |  |  | 1 | 1 |
| **7** |  |  |  |  |  |  |  |  |  |  |
| **8** |  |  |  |  |  |  |  |  |  |  |
| **9** |  |  |  |  |  |  |  |  |  |  |
| **10** |  |  |  |  |  |  |  |  |  |  |
| **11** |  |  |  | 1 |  |  |  |  |  |  |
| **12** |  |  |  |  |  |  |  |  | 1 |  |
| **13** |  |  | 1 |  |  |  |  |  |  |  |
| **14** |  |  |  | 1 |  |  |  |  |  |  |
| **15** |  |  |  | 1 | 1 | 1 |  |  |  |  |
| **16** |  |  |  |  | 1 |  |  |  |  |  |
| **17** |  |  | 2 | 3 | 1 | 1 |  |  |  |  |
| **18** |  |  | 2 | 6 | 2 |  |  |  |  |  |
| **19** |  |  |  | 5 | 2 |  |  |  |  |  |
| **20** |  |  | 2 | 2 |  |  |  |  |  |  |
| **21** |  | 1 | 3 | 1 |  |  |  |  |  |  |
| **22** |  | 1 | 1 |  |  |  |  |  |  |  |
| **23** |  |  |  |  |  |  |  |  |  |  |
| **24** | 1 | 1 | 1 |  |  | 1 |  |  |  |  |

**G.**

|  | **Agar dilution (mg/L)** | | | | | | | | | | | |
| --- | --- | --- | --- | --- | --- | --- | --- | --- | --- | --- | --- | --- |
| **Diameter (mm)** | **0.25** | **0.5** | **1** | **2** | **4** | **8** | **16** | **32** | **64** | **128** | **256** | **≥ 512** |
| **6** |  |  |  |  |  |  |  |  |  |  | 1 | 4 |
| **7** |  |  |  |  |  |  |  |  |  |  |  |  |
| **8** |  |  |  |  |  |  |  |  |  |  |  |  |
| **9** |  |  |  |  |  |  |  |  |  |  |  |  |
| **10** |  |  |  |  |  |  |  |  | 1 |  |  |  |
| **11** |  |  |  |  |  |  |  |  |  |  |  |  |
| **12** |  |  |  |  |  |  |  |  |  |  |  |  |
| **13** |  |  |  |  |  |  |  |  | 1 |  |  |  |
| **14** |  |  |  |  |  |  |  |  | 1 |  |  |  |
| **15** |  |  |  |  |  |  |  |  |  | 1 |  |  |
| **16** |  |  |  |  |  |  |  |  |  |  |  |  |
| **17** |  |  |  |  |  |  |  |  |  |  |  |  |
| **18** |  |  |  |  |  |  |  |  |  |  |  |  |
| **19** |  | 1 |  |  |  |  |  |  |  |  |  |  |
| **20** |  |  | 2 |  |  |  |  |  |  |  |  |  |
| **21** |  |  |  |  |  |  |  |  |  |  |  |  |
| **22** |  |  |  |  |  |  |  |  |  |  |  |  |
| **23** |  |  |  |  |  |  |  |  |  |  |  |  |
| **24** |  | 1 |  |  |  |  | 1 |  |  |  |  |  |
| **25** |  | 1 | 2 | 1 |  |  |  |  |  |  |  |  |
| **26** |  |  | 1 | 1 |  |  |  |  |  |  |  |  |
| **27** |  | 2 | 4 |  | 1 |  |  |  |  |  |  |  |
| **28** |  |  |  |  |  |  |  |  |  |  |  |  |
| **29** | 1 | 2 | 1 | 1 |  |  |  |  |  |  |  |  |
| **30** |  | 2 | 2 | 2 | 1 | 1 |  |  |  |  |  |  |
| **31** |  | 1 | 1 |  |  |  |  |  |  |  |  |  |
| **32** |  |  |  |  |  |  |  |  |  |  |  |  |
| **33** |  | 1 | 1 |  |  |  |  |  |  |  |  |  |
| **34** |  |  |  |  |  |  |  |  |  |  |  |  |
| **35** | 1 | 1 |  |  |  |  |  |  |  |  |  |  |

**Table S7.** Mecillinam agar dilution MIC against disc diffusion for *Citrobacter koseri* (A.), *Enterobacter cloacae* complex (B.), *Escherichia coli* (C.), *Klebsiella aerogenes* (D.), *Klebsiella oxytoca* (E.), *Klebsiella pneumoniae* (F.), and *Proteus mirabilis* (G.) strains.

The black lines designate the breakpoint used.

**A.**

|  | **Agar dilution (mg/L)** | | | | | | | | | | | | |
| --- | --- | --- | --- | --- | --- | --- | --- | --- | --- | --- | --- | --- | --- |
| **Diameter (mm)** | **0.06** | **0.12** | **0.25** | **0.5** | **1** | **2** | **4** | **8** | **16** | **32** | **64** | **128** | **≥ 256** |
| **6** |  |  |  |  |  |  |  |  |  |  |  |  | 1 |
| **7** |  |  |  |  |  |  |  |  |  |  |  |  |  |
| **8** |  |  |  |  |  |  |  |  |  |  |  |  |  |
| **9** |  |  |  |  |  |  |  |  |  |  |  |  |  |
| **10** |  |  |  |  |  |  |  |  |  |  |  |  |  |
| **11** |  |  |  |  |  |  |  |  |  |  |  |  |  |
| **12** |  |  |  |  |  |  |  |  |  |  |  |  |  |
| **13** |  |  |  |  |  |  |  |  |  |  |  |  |  |
| **14** |  |  |  |  |  |  |  |  |  |  |  |  |  |
| **15** |  |  |  |  |  |  |  |  |  |  |  |  |  |
| **16** |  |  |  |  |  |  |  |  |  |  |  |  |  |
| **17** |  |  |  |  |  |  |  |  |  |  |  |  |  |
| **18** |  |  |  |  |  |  |  |  |  |  |  |  |  |
| **19** |  |  |  |  |  |  |  |  |  |  |  |  |  |
| **20** |  |  |  |  |  |  |  |  |  |  |  |  |  |
| **21** |  |  | 1 |  |  |  |  |  |  |  |  |  | 1 |
| **22** |  | 2 | 1 |  |  |  |  |  |  |  |  |  | 1 |
| **23** |  | 2 | 1 |  |  |  |  |  |  |  |  |  |  |
| **24** |  |  |  | 1 |  |  |  |  |  |  |  |  |  |
| **25** | 3 | 3 | 4 |  |  |  |  |  |  |  |  |  |  |
| **26** | 1 | 7 | 1 | 1 |  |  |  |  |  |  |  |  |  |
| **27** |  | 7 | 1 |  |  |  |  |  |  |  |  |  |  |
| **28** | 1 | 2 | 1 |  |  |  |  |  |  |  |  |  |  |
| **29** | 2 | 2 | 1 |  |  |  |  |  |  |  |  |  |  |
| **30** |  |  |  | 1 |  |  |  |  |  |  |  |  |  |

**B.**

|  | **Agar dilution (mg/L)** | | | | | | | | | | | |
| --- | --- | --- | --- | --- | --- | --- | --- | --- | --- | --- | --- | --- |
| **Diameter (mm)** | **0.12** | **0.25** | **0.5** | **1** | **2** | **4** | **8** | **16** | **32** | **64** | **128** | **≥ 256** |
| **10** |  |  |  |  |  |  |  |  |  |  |  |  |
| **11** |  |  |  |  |  | 1 |  |  |  |  |  |  |
| **12** |  |  |  |  |  |  | 1 | 1 |  |  |  |  |
| **13** |  |  |  |  |  |  | 2 |  |  |  |  |  |
| **14** |  |  |  |  |  |  |  |  |  |  |  |  |
| **15** |  |  |  |  |  |  |  |  |  |  |  |  |
| **16** |  |  |  |  |  |  |  |  |  |  |  |  |
| **17** |  |  |  |  | 1 |  |  |  |  |  |  |  |
| **18** |  |  |  | 2 | 1 |  |  |  |  |  |  |  |
| **19** |  |  |  |  |  |  |  |  |  |  |  |  |
| **20** |  |  | 1 | 1 |  |  |  |  |  | 1 |  |  |
| **21** |  |  | 1 |  |  |  |  |  |  |  |  | 1 |
| **22** |  |  | 1 | 2 |  |  |  |  |  |  |  |  |
| **23** |  |  |  | 2 |  |  |  |  |  |  |  |  |
| **24** |  | 3 | 2 | 1 |  |  |  |  |  |  |  |  |
| **25** |  | 4 | 6 | 2 |  |  |  |  |  |  |  |  |
| **26** | 1 | 2 |  |  |  |  |  |  |  |  |  |  |
| **27** |  | 2 | 2 |  |  |  |  |  |  |  |  |  |
| **28** | 1 | 2 |  | 1 |  |  |  |  |  |  |  |  |
| **29** |  | 1 |  |  |  |  |  |  |  |  |  |  |
| **30** | 2 |  |  |  |  |  |  |  |  |  |  |  |

**C.**

|  | **Agar dilution (mg/L)** | | | | | | | |
| --- | --- | --- | --- | --- | --- | --- | --- | --- |
| **Diameter (mm)** | **0.12** | **0.25** | **0.5** | **1** | **2** | **4** | **8** | **16** |
| **6** |  |  |  |  |  | 1 |  |  |
| **7** |  |  |  |  |  |  |  |  |
| **8** |  |  |  |  |  |  |  |  |
| **9** |  |  |  |  |  |  |  |  |
| **10** |  |  |  |  |  |  |  |  |
| **11** |  |  |  |  | 1 |  |  |  |
| **12** |  |  |  |  |  |  |  |  |
| **13** |  |  |  |  |  |  |  |  |
| **14** |  |  | 1 | 1 | 1 | 1 |  |  |
| **15** |  |  |  | 2 |  |  |  |  |
| **16** |  |  | 1 | 3 | 1 |  |  |  |
| **17** |  |  |  |  |  |  |  |  |
| **18** |  |  |  |  |  |  |  |  |
| **19** |  |  | 1 | 1 |  |  |  |  |
| **20** |  |  | 1 |  |  |  |  |  |
| **21** |  | 1 | 1 |  |  |  |  |  |
| **22** |  | 3 |  |  |  |  |  |  |
| **23** |  | 1 |  |  |  |  |  |  |
| **24** | 1 | 3 | 2 |  |  |  |  |  |
| **25** | 1 | 2 |  |  |  |  |  |  |
| **26** | 2 | 3 |  |  |  |  |  |  |
| **27** | 2 | 3 |  |  |  |  |  |  |
| **28** | 4 | 1 |  |  |  |  |  |  |
| **29** | 1 | 1 |  |  |  |  |  |  |
| **30** |  |  | 1 |  |  |  |  |  |

**D.**

|  | **Agar dilution (mg/L)** | | | | | | | | | | |
| --- | --- | --- | --- | --- | --- | --- | --- | --- | --- | --- | --- |
| **Diameter (mm)** | **0.25** | **0.5** | **1** | **2** | **4** | **8** | **16** | **32** | **64** | **128** | **≥ 256** |
| **6** |  |  |  |  |  |  |  |  |  |  | 1 |
| **7** |  |  |  |  |  |  |  |  |  |  |  |
| **8** |  |  |  |  |  |  |  |  |  |  |  |
| **9** |  |  |  |  |  |  |  |  |  |  |  |
| **10** |  |  |  |  |  |  |  |  |  |  |  |
| **11** |  |  |  |  |  |  |  |  |  |  |  |
| **12** |  |  |  |  |  |  |  |  |  |  |  |
| **13** |  |  |  |  |  | 1 |  |  |  |  |  |
| **14** |  |  |  |  |  |  |  |  |  |  |  |
| **15** |  |  |  |  |  |  |  |  |  |  |  |
| **16** |  |  |  |  |  |  |  |  |  |  |  |
| **17** |  | 1 |  |  |  |  |  |  |  |  |  |
| **18** |  |  |  | 1 |  |  |  |  |  |  |  |
| **19** |  | 1 | 1 |  |  |  |  |  |  |  |  |
| **20** |  | 3 |  |  |  |  |  |  | 1 |  |  |
| **21** |  |  | 3 |  |  |  | 1 |  |  |  |  |
| **22** |  | 3 | 4 |  |  |  |  | 1 |  |  |  |
| **23** |  | 5 | 3 |  |  |  |  |  |  |  |  |
| **24** |  | 9 | 2 | 1 |  |  |  |  |  |  |  |
| **25** |  | 4 |  |  |  |  |  |  |  |  |  |
| **26** |  |  |  |  |  |  |  |  |  |  |  |
| **27** | 1 | 1 |  |  |  |  |  |  | 1 |  |  |

**E.**

|  | **Agar dilution (mg/L)** | | | | | | | | | |
| --- | --- | --- | --- | --- | --- | --- | --- | --- | --- | --- |
| **Diameter (mm)** | **0.25** | **0.5** | **1** | **2** | **4** | **8** | **16** | **32** | **64** | **128** |
| **6** |  |  |  | 1 |  |  |  |  |  | 1 |
| **7** |  |  |  |  |  |  |  |  |  |  |
| **8** |  |  |  |  |  |  |  |  |  |  |
| **9** |  |  |  |  |  |  |  |  |  |  |
| **10** |  |  |  |  |  |  |  |  |  |  |
| **11** |  |  |  |  |  |  | 1 |  |  |  |
| **12** |  |  |  |  | 1 |  |  |  |  |  |
| **13** |  |  |  |  | 1 |  | 1 |  |  |  |
| **14** |  |  |  | 1 |  |  |  |  |  |  |
| **15** |  |  |  |  | 1 |  |  |  |  |  |
| **16** |  | 1 |  |  |  |  |  |  |  |  |
| **17** |  |  | 1 |  | 1 |  |  |  |  |  |
| **18** | 2 | 1 |  |  |  |  |  |  |  |  |
| **19** |  | 1 |  |  |  |  |  |  |  |  |
| **20** | 2 | 6 |  |  |  |  |  |  |  |  |
| **21** | 2 | 3 |  |  | 1 |  |  |  |  |  |
| **22** | 1 | 2 | 1 |  |  |  |  |  |  |  |
| **23** | 1 | 3 |  | 1 |  |  |  |  |  |  |
| **24** | 3 |  |  |  |  |  |  |  |  |  |
| **25** | 6 | 1 |  |  |  |  |  |  |  |  |
| **26** | 1 | 1 |  |  |  |  |  |  |  |  |

**F.**

|  | **Agar dilution (mg/L)** | | | | | | | | | | | |
| --- | --- | --- | --- | --- | --- | --- | --- | --- | --- | --- | --- | --- |
| **Diameter (mm)** | **0.125** | **0.25** | **0.5** | **1** | **2** | **4** | **8** | **16** | **32** | **64** | **128** | **≥ 256** |
| **6** |  |  |  | 1 |  |  |  | 1 |  |  |  | 1 |
| **7** |  |  |  |  |  |  |  |  |  |  |  |  |
| **8** |  |  |  |  |  |  |  |  |  |  |  |  |
| **9** |  |  |  |  |  |  |  | 1 |  |  |  |  |
| **10** |  |  |  |  |  |  | 1 |  |  |  |  |  |
| **11** |  |  |  |  |  |  | 1 |  |  |  |  |  |
| **12** |  |  |  |  |  |  |  |  |  |  |  |  |
| **13** |  |  |  |  |  | 1 |  |  |  |  |  |  |
| **14** |  |  | 1 |  |  |  |  |  |  |  |  |  |
| **15** |  |  |  |  |  |  |  |  |  |  |  |  |
| **16** |  |  | 1 |  | 1 |  |  |  |  |  |  |  |
| **17** |  |  |  |  |  |  |  |  |  |  |  |  |
| **18** |  |  | 1 | 1 |  |  |  |  |  |  |  |  |
| **19** |  | 1 |  | 3 |  | 1 |  |  |  |  |  |  |
| **20** |  | 2 | 2 |  |  |  |  |  |  |  |  |  |
| **21** |  | 2 |  |  |  |  |  |  |  |  |  |  |
| **22** |  | 3 |  |  |  |  |  |  |  |  |  |  |
| **23** |  | 1 |  |  |  |  |  |  |  |  |  |  |
| **24** | 1 | 6 | 1 |  |  |  |  |  |  |  |  |  |
| **25** |  | 7 | 1 |  |  |  |  |  |  |  |  |  |
| **26** | 1 | 1 | 1 |  |  |  |  |  |  |  |  |  |
| **27** |  |  |  |  |  |  |  |  |  |  |  |  |
| **28** | 1 | 1 | 1 |  |  |  |  |  |  |  |  |  |

**G.**

|  | **Agar dilution (mg/L)** | | | | | | | | | | | |
| --- | --- | --- | --- | --- | --- | --- | --- | --- | --- | --- | --- | --- |
| **Diameter (mm)** | **0.125** | **0.25** | **0.5** | **1** | **2** | **4** | **8** | **16** | **32** | **64** | **128** | **≥ 256** |
| **6** |  |  |  |  |  | 1 | 2 | 1 | 1 | 1 | 2 | 3 |
| **7** |  |  |  |  |  |  |  |  |  |  |  |  |
| **8** |  |  |  |  |  |  |  |  |  |  |  |  |
| **9** |  |  |  |  |  | 1 |  |  |  |  |  |  |
| **10** |  |  |  |  |  |  |  |  |  |  |  |  |
| **11** |  |  |  |  |  |  |  |  |  |  |  |  |
| **12** |  |  |  |  |  |  |  |  |  |  |  | 1 |
| **13** |  |  |  |  |  |  |  |  |  |  |  |  |
| **14** |  |  |  |  |  |  |  |  |  |  |  | 1 |
| **15** |  |  |  |  |  |  |  |  | 1 |  |  |  |
| **16** |  |  |  |  |  |  |  |  |  |  |  | 1 |
| **17** |  |  |  |  |  |  |  |  |  |  |  |  |
| **18** |  |  |  |  |  |  |  |  |  |  |  |  |
| **19** |  |  |  |  |  |  |  |  |  |  |  |  |
| **20** |  |  | 3 | 3 | 2 | 2 |  |  | 1 |  |  |  |
| **21** |  |  | 1 |  | 1 |  |  |  |  |  |  | 1 |
| **22** |  | 3 | 3 | 1 | 1 | 1 | 1 |  |  |  |  |  |
| **23** |  | 1 | 1 | 1 | 1 |  |  |  |  |  |  |  |
| **24** | 2 |  |  |  |  |  |  |  |  |  |  |  |
| **25** |  |  |  |  |  |  |  |  |  |  |  |  |
| **26** |  | 2 |  |  |  | 1 |  |  |  |  |  |  |
| **27** |  |  | 1 |  |  |  |  |  |  |  |  |  |
| **28** |  |  |  |  |  |  |  |  |  |  |  |  |
